# Evaluation of the utilization of external radiotherapy in the treatment of localized prostate cancer in Andalusia, Spain

**DOI:** 10.1186/s13014-015-0572-8

**Published:** 2015-12-30

**Authors:** José Expósito, Isabel Linares, Isabel Castillo, Miguel Martínez, Pilar Vargas, Ismael Herruzo, José Antonio Medina, Amalia Palacios, Eloísa Bayo, Francisco Peracaula, Javier Jaén, José Antonio Sánchez, María José Ortiz

**Affiliations:** Radiotherapy and Oncology Department, Granada General Hospital, Avda. fuerzas Armadas, s/n, 18014 Granada, Spain; Radiotherapy and Oncology Department, Granada General Hospital, Calle Dr. Oloriz, 16, 18012 Granada, Spain; Radiotherapy and Oncology Department, Jaén General Hospital, Avda. del Ejército Español, 10, 23007 Jaén, Spain; Radiotherapy and Oncology Department, Carlos Haya University Hospital, Avenida Carlos Haya, s/n, 29010 Málaga, Spain; Radiotherapy and Oncology Department, Virgen de la Victoria University Hospital, Campus de Teatinos, s/n, 29010 Málaga, Spain; Radiotherapy and Oncology Department, Reína Sofía University Hospital, Avda. Menéndez Pidal, s/n, 14004 Córdoba, Spain; Radiotherapy and Oncology Department, Juan Ramón Jiménez Hospital, Avda. de la Orden, 21005 Huelva, Spain; Radiotherapy and Oncology Department, Punta de Europa Algeciras Hospital, Carr Getares, S/N, 11207 Algeciras, Cádiz, Spain; Radiotherapy and Oncology Department, Puerta del Mar University Hospital, Av. Ana de Viya, 21, 11009 Cádiz, Spain; Radiotherapy and Oncology Department, Virgen Macarena University Hospital, Avda. Dr Fedriani, 3, 41071 Sevilla, Spain; Radiotherapy and Oncology Department, Virgen del Rocío University Hospital, Avda. Manuel Siurot, s/n, 41013 Sevilla, Spain

**Keywords:** Prostate cancer, Radiotherapy, Variability, Use

## Abstract

**Background:**

Around 27,000 new cases of prostate cancer are diagnosed every year in Spain and 5400 die from this disease. Radiotherapy (RT), alone or combined, has proven to be effective as initial treatment in patients with localized disease. Our objective was to evaluate the use of external beam RT (EBRT) in our region, comparing the indication rate and irradiation rate and examining variability in its application among hospitals.

**Methods:**

We conducted a review of RT guidelines and indication studies for prostate cancer (% expected irradiation). Data were gathered from all twelve public healthcare centers in Andalusia (Spain) on RT-treated prostate cancer patients during 2013 (% actual irradiation) and from nine of the centers on RT discharge reports. Information was classified according to type of hospital, tumor risk category and RT treatment (technique, dosage, volume, toxicity).

**Results:**

The estimated RT rate was 67 % (1289/1917), 43 % were aged > 70 years, 44.7 % had ECOG performance status of 0); 44.7 % had high-risk tumors; 57 % underwent RT associated with hormone therapy; 70 % of patients receiving RT were treated with 3D planning (30 % IGRT); and doses were 70–76 Gy in 70 % of cases and >76 Gy in 10.7 %. Acute gastrointestinal and genitourinary toxicities were < grade 2 in 79 and 89 % of patients, respectively. An irradiation rate significantly below the mean for the study was found in four provinces. There was a significant difference among provinces in the distribution of risk groups.

**Conclusions:**

Underutilization of EBRT was estimated to be around 30 % in prostate cancer patients, with an elevated variability in irradiation rates among hospitals related to differences in available technology and in the distribution of patients with different risk levels. These data should be a matter of concern to regional health managers, given the negative and measurable impact on the survival of patients.

## Background

Prostate cancer (PrC) is the second most frequent tumor in males in the industrialized world, with an estimated standardized rate (European population) of 62.5 × 10^5^ and a mortality of 8.8 × 10^5^ (27,852 and 5481 cases, respectively). In Andalusia, a region in southern Spain with 8.4 million inhabitants, PrC represents a total of 3280 new cases/year and 1250 deaths/year [1–3]. An increase in this disease has been detected over the past few decades, probably in part due to a greater use of prostate-specific antigen (PSA) testing, without ruling out the influence of unknown factors [4].

This higher incidence is associated with an increase in the number of patients with localized disease (gland and surrounding anatomical area) at diagnosis, permitting greater disease control by surgery and radiotherapy (RT). The classification in risk degrees for localized disease and prognostic algorithms enable local treatment options to be offered with or without hormone therapy, which is currently the standard treatment [5]. Despite the lack of randomized clinical trials to compare among prostatectomy, external beam RT (EBRT), or brachytherapy for localized disease, there is international consensus on the role of EBRT in the local treatment of PrC, as reflected in the guidelines of scientific societies [6]. Recent advances have allowed 3D planning techniques to be used to deliver high irradiation doses with lower toxicity. EBRT techniques using guided imaging and modulated dose intensity, although not available in all RT departments, permit a superior dose distribution that confers higher treatment safety with lower toxicity and is expected to achieve greater control of the disease. It also allows the delivery of larger doses through hypofractionation, shortening treatment times. These technological improvements have led to the application of a variety of RT regimens for these tumors [7].

Medical practice variability (MPV) can have a negative impact on health outcomes and cost-effectiveness [8]. MPV has been attributed to differences in the availability (number or geographic proximity) of resources and in the practice of professionals [9]. Previous research in our setting (VARA II), based on a review of clinical records and treatment reports and considering recommended indications, showed that the underutilization of EBRT in lung cancer had a negative impact on patient outcomes [10–12].

The objectives of the present study were to evaluate the use of EBRT as initial treatment for patients with PrC in hospitals in a region of Southern Spain, comparing expected with actual irradiation rates and examining the variability in its application among participating centers and associated factors.

## Methods

A longitudinal retrospective study was conducted between January 1 and December 31 2013 in the 12 public hospitals with RT facilities in Andalusia, an autonomous community in southern Spain with 8.4 × 10^6^ inhabitants. These centers are distributed among the 8 provinces that form the autonomous community, ensuring coverage of the whole population.

We gathered data on all patients with non-metastatic PrC of any histological type and degree of risk whose initial treatment was EBRT. This information was obtained from the clinical management computer systems associated with the RT equipment (Varis®, Lantis®, Impac®, Mosaiq® networks, etc.). Demographic data were gathered from the Spanish National Institute of Statistics (http://www.ine.es) [13], while information on cancer incidence and distribution among histological types and stages were extrapolated using data from the 2010 National Prostate Cancer Registry [14] and Carlos III Health Institute (Madrid) [15]. The irradiation rate was obtained by calculating the percentage of irradiated cases with respect to the total number of diagnosed cases, examining the variability by hospital/catchment area. Expected irradiation rates were based on the studies by Tyldesley S et al. and Delaney et al. [16, 17], which define the proportion of patients at each risk level for whom RT (external beam or brachytherapy) would be indicated. Data were gathered from the clinical records on the general state of patients as measured with the ECOG Performance Status (PS) score.

Acute toxicity data were measured following the EORTC/RTOG criteria.

EBRT application variability was studied by analyzing the cases treated in nine of the participating centers, gathering data on the characteristics of the hospital (treatment units/professionals), patients, therapies, and tumors (histology, stage) and on the RT modalities and regimens (doses and combination with hormone therapy [HT]) (Table 1). We excluded patients treated after surgery or after biochemical recurrence and those receiving palliative treatment for bone metastases. Information on each patient was extracted from treatment discharge reports by trained personnel under the supervision of the research team.

**Table 1:** Study variables

Tumor	Histology, classification (low, intermediate, and high risk), diagnosis date, and Performance Score
RT treatment	Dates of start and end of RT, volume, total dose, dose fraction, technique, energy, acute toxicity, interruptions and cause, associated hormone therapy: duration and drugs.

### Ethical approval

The study has been approved by the provincial Biomedical Research Ethics Committee and has therefore been performed in accordance with the ethical standards laid down in the 1964 Declaration of Helsinki. All patients gave their informed consent prior to their inclusion in the study. Details that might disclose the identity of the subjects under study have been omitted.

### Statistical analysis

A descriptive analysis was performed, calculating central tendency and dispersion statistics (mean, median, interquartile range, standard deviation, 95 % confidence interval). The chi-square (χ2) test was used to compare qualitative variables and exact Fisher’s test for binary variables. Relationships among quantitative variables were studied by using Pearson’s correlation coefficient (or the non-parametric Kendall’s Tau-b and Spearman tests) and linear regression analysis. A two-sided *p* < 0.05 was considered significant. SPSS version 12.0 was used for statistical analyses.

## Results

### Irradiation rate

Among patients with PrC, 88 % are expected to have localized disease at their diagnosis, estimating an optimal irradiation rate of 58 % with EBRT and 9 % with brachytherapy [18]. Accordingly, 1917 of the present series of PrC patients would be expected to undergo RT, 41 % of low-risk (*n* = 1183), 25 % of intermediate-risk (721), and 31 % of high-risk (*n* = 894) patients. In fact, only 1160 of the patients received EBRT and 129 brachytherapy, i.e., 67 % of the expected total.

### EBRT type and variability

As shown in Table 2, the type of EBRT treatment was recorded in 609 (52.50 %) of the 1160 irradiated patients; 43 % were aged > 70 years; 47.6 % were in a good general state (PS = 0); 44.7 % had high-risk, 32 % intermediate-risk, and 23 % low-risk disease. 57 % received RT combined with HT;67 % of patients received a combined RT-HT therapy (we are excluding patients with BQT (14.4 %). On the other hand, the percentage of patients receiving a combined RT-HT stratified by risk group was as follows: 23 % for low-risk patients, 71.5 % for intermediate-risk patients, and 89.5 % for high-risk patients. Excluding those patients treated with BQT as monotherapy, 72.5 % of patients were irradiated using 3D planning techniques; 70.7 % received 76 Gy (1.8–2 Gy/fr) and 10.8 % were prescribed higher doses than 76 Gy (1.8–2Gy/fr). 18.5 % received less than 76 Gy but using a hypo-fractionation scheme with doses-per-fraction > 2 Gy. Volumes were centered on prostate gland in 64 % and seminal vesicles in 23 %. Only 12.9 % received RT to the lymph nodes; this might be explained by the fact that 46.3 % of patients were both high-risk and aged 70 years or more, indicating clinicians reluctance and true concern to prescribed lymph nodes RT in patients identified as frail, elderly patients, in order to avoid a live-threatening toxicity. Acute intestinal and genitourinary toxicities were < grade 2 in 79 and 89 % of patients, respectively.

**Table 2:** Main variables characteristics

Age (ranges)	40–60	77 (12.6 %)
	61–70	262 (43 %)
	>70	265 (43.5 %)
	Unknown	5 (0.8 %)
Performance score	0	290 (47.6 %)
	1	72 (11.8 %)
	2	1 (0.2 %)
	3	1 (0.2 %)
	Unknown	245 (40.2 %)
Risk (D’Amico)	Low	140 (23 %)
	Intermediate	195 (32 %)
	High	272 (44.7 %)
	Unknown	2 (0.3 %)
Treatment	RT	85 (14 %)
	RT + HT	348 (57.1 %)
	Cx + RT	87 (14.3 %)
	BQ	88 (14.4 %)
	Unknown	1 (0.2 %)
RT technique	3D	369 (60.6 %)
	IGRT	140 (23.0 %)
	BQ	88 (14.4 %)
	Unknown	12 (2.0 %)
RT dose (Gy)	<76	96 (15.8 %)
	76	367 (60.3 %)
	>76	56 (9.2 %)
	Unknown	2 (0.3 %)
	BQ	88 (14.4 %)
Volume^a^	Prostate	331 (63.5 %)
	+ Vesicles	119 (22.8 %)
	+ Lymph nodes	67 (12.9 %)
	Unknown	4 (0.8 %)
Toxicity^b^	GI 0	218 (41.8 %)
	1	175 (33.6 %)
	2	86 (16.5 %)
	3	2 (0.4 %)
	Unknown	40 (7.7 %)
	GU 0	432 (82.9 %)
	1	38 (7.3 %)
	2	14 (2.7 %)
	3	1 (0.2 %)
	Unknown	36 (6.9 %)

*RT* radiotherapy, *HT* hormone therapy, *Cx* surgery, *IGRT* image-guided radiation therapy, *GI* gastrointestinal, *GU* genitourinary

^a^Treatment volume excluding Brachytherapy

^b^Toxicity excluding Brachytherapy

Irradiation dosages and volumes were significantly correlated with risk levels (Table 3). A high risk was associated with doses >76 Gy (*p* < 0.003) and with the inclusion of vesicles and lymph nodes in the target volume (*p* < 0.001). However, neither intestinal nor urinary toxicity was correlated with the total dose, treated volume, or degree of risk.

**Table 3:** Relationship of risk with variables

Variables	Pearson’s Chi^2^ test	P	Comment
Risk– total dose	15.738	0.003	Higher risk, higher dose
Risk – dose fraction	30.746	0.000	
Risk – volume	121.225	0.000	Higher risk, higher volume
Risk – urinary toxicity	8.602	0.197	No relationship
Risk –gastrointestinal toxicity	3.608	0.730	No relationship

**Fig. 1:**
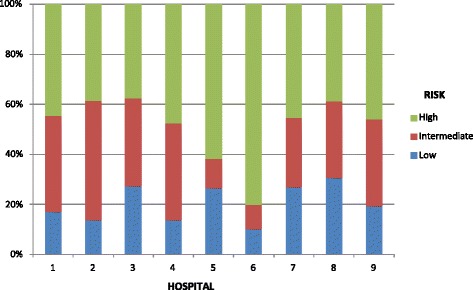
Distribution of degree of risk by hospital

The participating hospital centers showed significant differences in the irradiation rate. As shown in Table 4, a significantly lower number of patients underwent RT than expected in hospitals 3, 6, 7, and 8, whereas a significantly higher number than expected was treated in hospital 4 (Table 4). There was also a difference in the distribution of risk groups (Fig. 1), with a significantly higher proportion of high-risk patients in hospitals 5 and 6 than in the others. Concerning the analysis of other variables, such as total dose, dose per fraction and technique, some differences between hospitals were found. Regarding total dose most centers prescribed 76 Gy in 70–90 % of cases. Only in hospital 6 and 7 was a total dose < 76 Gy prescribed, but using an hypofractionated scheme with doses per fraction > 2Gy; it should be pointed out that these hospitals had at their disposal IGRT systems. Hospital 3 was the one who presented the highest percentage of patients treated with total doses > 76 Gy. Regarding the techniques used in each center, hospital 1, 2 and 3 lacked any IGRT system. Hospital 1, 3 and 5 did not have BQT and the percentage of patients being treated with this technique (referring patients to other centers) was lower than the rest of centers.

**Table 4:** Distribution of expected and observed cases by center

Hospital by province	Population	Expected Frequency	Expected Rate (cases/10000 inhabitants)	95 % CI		Observed Frequency	Observed rate (cases/10000 inhabitants)	95 % CI	
				low	high			low	high
3	518 687	103	1.99	1.60	2.37	83	1.60	1.26	1.94
11	698 601	130	1.86	1.54	2.18	108	1.55	1.25	1.84
8^a^	700 570	139	1.98	1.65	2.31	90	1.28	1.02	1.55
6^a^	798 580	158	1.98	1.67	2.29	258	3.23	2.84	3.63
2,7	918 382	182	1.98	1.69	2.27	185	2.01	1.72	2.31
4,5^a^	1 238 291	246	1.99	1.74	2.24	44	0.36	0.25	0.46
1,10^a^	1 619 497	321	1.98	1.77	2.20	211	1.30	1.13	1.48
9^a^	1 940 027	385	1.98	1.79	2.18	300	1.55	1.37	1.72

^a^Significant differences between observed and expected values

## Discussion

Knowledge of irradiation rates defines the contribution of radiation oncologist to the management of cancer patients and is important for the allocation of RT resources [19, 20]. Various methods have been proposed to estimate the irradiation rate as accurately as possible, following benchmarking [21], expert, or evidence-based criteria [22]. The most updated approach [18] was used in this study of hospitals in a southern Spanish region, which found that EBRT was not administered to almost three out of every ten patients who could have been expected to receive it.

There are various possible explanations for this apparent underutilization of RT. Urologists, who initiate the PrC diagnostic process, may be more inclined to support surgery rather than EBRT, especially in low- and intermediate-risk patients, who represented a large proportion of the present series. Thus it would be important to develop multidisciplinary teams, in order to better assign patients who might benefit from a RT treatment. We highlight the finding that image-guided RT, considered the most effective technique [23], was only possible in 30 % of the patients, concentrated in the three centers possessing this facility at the time of the study. Despite efforts to improve the situation over the past few years, the availability of this technology remains suboptimal in Andalusia [3, 24]. Technological limitations may also account for the low total dosages and little utilization of doses > 2 Gy per fraction (hypofractionation), a widespread approach in PrC treatment, although it may also reflect certain reservations among professionals about the application and safety of less standard techniques [11]. The low dosages may in part explain the low toxicity levels, but further research is required to establish their effect on final patient outcomes and compare these with other reports [25]. Another technological limitation in Andalusia was that only 4 centers were able to offer a brachytherapy treatment to patients having a low-risk which, as it is already well established in the literature, is an alternative to other treatments [26]. Another important issue in terms of variability would be the importance of active vigilance in a subset of patients with low-risk; even if this active surveillance is a recommended option [27] for this group of patients, especially the older one among them, in Andalusia is not still an option currently offered to our patients. At least, authors of this article have no record of it.

Besides differences in equipment, the elevated variability among hospitals would also be related to variations in the distribution of patients with different risk levels. Thus, hospitals 5 and 6 had a much larger percentage of high-risk patients, which would imply a more frequent use of surgery as local treatment.

Besides all of these causes mentioned above, we have to conclude that an important part of the variability found within Andalusia is due to an intrinsic variability between physicians.

Study limitations include the lack of data on treatment regimens from three hospitals and the non-participation of private centers, although these only represent around i5–10 % of PrC patients in our region. However, relevant nformation was obtained on the inadequacy and variability of RT utilization, making the case for a greater prioritization of scarce resources to remedy this situation [28].

Given the impediments to conducting clinical trials on RT, further research is warranted to compare its utilization and outcomes in different cancer types among centers with varied technological resources [29, 30].

## Conclusions

In representative public hospitals from our region, radiotherapy was not delivered to around 30 % of prostate cancer patients who could benefit from this treatment. An elevated variability among centers in the irradiation rate was related to differences in risk distribution and the availability of high-performance radiation therapy. These data should be a matter of concern to regional health authorities, given the measurable negative impact on the survival of patients.

## Abbreviations

EBRT: external beam radiotherapy
HT: hormone therapy
MPV: medical practice variability
PrC: prostate cancer
PS: performance status
PSA: prostate-specific antigen
RT: radiotherapy
